# A biomechanical assessment of hydraulic ankle-foot devices with and without micro-processor control during slope ambulation in trans-femoral amputees

**DOI:** 10.1371/journal.pone.0205093

**Published:** 2018-10-05

**Authors:** Xuefei Bai, David Ewins, Andrew David Crocombe, Wei Xu

**Affiliations:** 1 National Research Center for Rehabilitation Technical Aids, Beijing, China; 2 Beijing Key Laboratory of Rehabilitation Technical Aids for Old-Age Disability, Beijing, China; 3 Key Laboratory of Human Motion Analysis and Rehabilitation Technology of the Ministry of Civil Affairs, Beijing, China; 4 Department of Mechanical Engineering Sciences, University of Surrey, Guildford, United Kingdom; 5 Gait Laboratory, Queen Mary’s Hospital, Roehampton, London, United Kingdom; Northwestern University, UNITED STATES

## Abstract

Slope ambulation is a challenge for trans-femoral amputees due to a relative lack of knee function. The assessment of prosthetic ankles on slopes is required for supporting the design, optimisation, and selection of prostheses. This study assessed two hydraulic ankle-foot devices (one of the hydraulic ankles is controlled by a micro-processor that allows real-time adjustment in ankle resistance and range of motion) used by trans-femoral amputees in ascending and descending a 5-degree slope walking, against a rigid ankle-foot device. Five experienced and active unilateral trans-femoral amputees performed ascending and descending slope tests with their usual prosthetic knee and socket fitted with a rigid ankle-foot, a hydraulic ankle-foot without a micro-processor, and a hydraulic ankle-foot with a micro-processor optimised for ascending and descending slopes. Peak values in hip, knee and ankle joint angles and moments were collected and the normalcy Trend Symmetry Index of the prosthetic ankle moments (as an indication of bio-mimicry) were calculated and assessment. Particular benefits of the hydraulic ankle-foot devices were better bio-mimicry of ankle resistance moment, greater range of motion, and improved passive prosthetic knee stability according to the greater mid-stance external knee extensor moment (especially in descending slope) compared to the rigid design. The micro-processor controlled device demonstrated optimised ankle angle and moment patterns for ascending and descending slope respectively, and was found to potentially further improve the ankle moment bio-minicry and prosthetic knee stability compared to the hydraulic device without a micro-processor. However the difference between the micro-processor controlled device and the one without a micro-processor does not reach a statistically significant level.

## Introduction

Walking on sloped surfaces is a common task in daily life. According to gait studies with able-bodied subjects, the knee joint has been found to be a major adaptor for slope ambulation as it provides additional flexion and extensor moment in the early and mid-stance phase compared to level ground walking [1–4]. Currently most trans-femoral amputees (TFAs) use passive prosthetic knees that do not actively provide extensor moment and are designed to be locked in nearly full extension from initial contact until the pre-swing phase. Therefore, it is normally more of a challenge for TFAs to walk on slopes compared to trans-tibial amputees (TTAs) [5]. With the development of prostheses and the needs of amputees, recently more gait studies have been carried out on the slope walking of TFAs [5, 6]. As the passive prosthetic knees need to maintain extension during most of stance, the prosthetic ankle joint is crucial to supplement lower limb movement. However, most studies on the slope ambulation of the TFAs have been associated with prosthetic knee development and assessment, with limited studies on prosthetic ankle function.

The hydraulic ankle-foot device is a passive single axis articulating prosthesis design that has been recently introduced and become commercially available for lower limb amputees. More recently, micro-processor controlled hydraulic ankles have been developed and the micro-processor allows the ankle resistance and range of motion (ROM) to be set with a mobile app at any time. The function of hydraulic ankles have been investigated in previous studies [7–14]. Most gait studies were carried out with TTAs and tests have been performed on level ground [8, 9, 11], slopes [12, 14] and general outdoor walking conditions (including slopes and stairs) [7]. Two studies involved TFAs and both level ground and cross-slope walking were investigated [11, 13]. It has been reported that the hydraulic ankle enables increased walking speed [9, 11], a smoother transfer of plantar centre of pressure [8, 11], a higher bio-mimicry of ankle resistance [13], and decreased peak internal stresses on the stump [7]. However, other research reported no significant differences in the torque at the distal end of the socket in TTAs during slope ambulation when using hydraulic ankle-foot devices compared with other ankle-foot designs [14]. The results of a questionnaire evaluation study (Seattle Prosthesis Evaluation Questionnaire) showed improved satisfaction from 9 amputees (3 unilateral TTAs, 3 bilateral and 3 unilateral TFAs) when using a hydraulic ankle-foot device compared with their standard devices [10]. To date, only one study assessed a micro-processor controlled hydraulic ankle and this was done with TTAs in descending a slope [12]. It was reported that compared with a fixed hydraulic ankle and a rubber ball-joint ankle-foot device, when the micro-processor was activated, there were significantly reduced prosthetic shank single-support mean rotation velocity, residual knee flexion, residual knee negative work, and greater negative prosthetic ankle work, which indicated that reduced biomechanical compensations were used when walking down slopes [12]. Overall, more studies on the effects of micro-processor controlled hydraulic ankle units in slope ambulation and the effect of a hydraulic ankle in TFAs in general are required to support the optimisation of prostheses design and selection of prosthetic components.

The pattern of the prosthetic ankle moment has been linked with the walking experience of amputees using a mathematical model and it was found that a prosthetic ankle moment pattern that is similar to (biomimicing) that of non-amputees could introduce a substantial decrease in stresses on the residual limb [15]. Therefore the quantified similarity between prosthetic ankle moment and biological ankle moment can be used to assess the prosthetic ankle-foot device. However, probably due to the lack of an appropriate quantification method, so far there has only been one study that has tried to use Trend Symmetry Index (TSI) to assess different types of prosthetic ankle-foot devices [13].

The first aim of this research was to investigate changes in kinematic and kinetic data of a hydraulic ankle in ascending and descending a 5-degree slope in TFAs in comparison to a rigid ankle. Based on the findings in a previous study [13], it was hypothesised that the hydraulic ankle would provide a better biomimetic ankle moment (more similar to the biological ankle moment pattern) and a greater ROM in slope walking compared with the rigid ankle. The second aim was to investigate the effects of a micro-processor controlled hydraulic ankle that allows customised adjustments in the adaption to uphill and downhill slope walking. It is hypothesised that the micro-processor controlled hydraulic ankle would provide further improved biomimetic ankle moments to adapt to the slope surface than the non micro-processor version. In addition, the hip and knee kinematics and kinetics on both sides can be affected by the change of prosthetic foot, with limited previous work on the investigation of the effect of hydraulic ankle-foot devices on the other lower limb joints in TFAs, the hip and knee angles and moments were also studied.

## Methods

### Subjects and prostheses

Amputee subjects were recruited by the prosthetist that cooperated with this research project. No contact was made between subjects and researchers unless the subjects meet the selection criteria and agree to participate. The recruited amputee participants met the following inclusion criteria: 1) Unilateral trans-femoral amputees who have finished their whole rehabilitation program; 2) Over the age of 18; 3) Participants have had a review with their prosthetist within two months prior to the data collection day and have no outstanding issues with the prosthesis fit or stump; 4) Participants mobility to be scored as level E or above using the SIGAM tool: "walks 50 metres or more without walking aids except to improve confidence in adverse terrain or weather" (or equivalent K-Levels K3 and K4); 5) Able to negotiate ramps without any additional walking aids. The exclusion criteria for an amputee participant includes: 1) Participants with visual, auditory or vestibular impairment that affects balance, walking or the ability to follow and respond to verbal instructions; 2) Participants with sensitive skin or dermatological problems; 3) Participants experiencing oedema at the stump; and 4) Participants who are recently, or are currently, involved in another similar research project studying the function of the prosthetic ankle. Non-amputee (NA) subjects were recruited from the population around the University of Surrey via invitation Email sent by the researcher. The non-amputee participants needed to be over 18 and willing to handle the experimental objects. The exclusion criteria for a non-amputee participant includes: 1) Participants with visual, auditory or vestibular impairment that affects balance, walking or the ability to follow and respond to verbal instructions; 2) Participants with known motor impairments or injuries that influence movement; 3) Participants needing mobility aids. Hard copies of the participant information sheet and consent form were sent to all subjects prior to the data collection. Written consent was collected from each subject before their first walking session.

Five TFA subjects (5 males, age: 42±17 years; weight with prostheses: 107±16 kg; height: 1.83±0.02 m; residual limb length measured from the anterior superior iliac spine to the distal end of the stump: 0.45±0.07 m) who walked actively with their prostheses on a daily basis participated in this research. Except for the prosthetic ankle-foot and the shank tube, participants used their own prosthetic components during the tests. The details of the subjects normally used prostheses are summarised in Table 1. Fourteen NA subjects (5 males and 9 females, age: 26±2 years, weight: 68±15 kg, height: 1.69±0.08 m) were investigated in the research to generate the mean biological ankle moment curve required for the normalcy TSI calculation. Ethical approval for this study was given by the UK NHS National Research Ethics Service Committee London (Bloomsbury).

**Table 1:** Details of the prostheses normally usedby TA subjects.

ID	Prosthetic side	Years using prostheses	Socket	Knee^a^	Foot^a^
TF1	R	13	Carbon outer and pelite inner	KX06	EchelonVT
TF2	L	22	Suction with ossur seal in liner	KX06	Elan
TF3	R	4	Sealin suction socket	Linx	Linx
TF4	R	28	Sealin suction socket	smart IP	Elan
TF5	L	5	Suction with ossur seal in liner	Linx	Linx

^a^ The brand of all prosthetic knees and feet was Endolite.

In the three prosthetic ankle-foot devices tested in this study, the structures below the “ankle” joint, including carbon toe and heel springs, are the same. One of the prosthetic feet (Esprit, Blatchford & Sons Ltd., Basingstoke, UK; labelled as FIX for this study) does not have an articular ankle joint. The other two feet have a hydraulic single axis articular joint that is adjustable in the ROM in the sagittal plane and in the resistance moment. One of the hydraulic feet (Elan, Blatchford & Sons Ltd., Basingstoke, UK; labelled as MPC-HY for this study) includes a microprocessor that could be controlled in real time by the user via their mobile phone through a Bluetooth connection. The setting of the valve adjuster in the other hydraulic foot (Echelon, Blatchford & Sons Ltd., Basingstoke, UK; labelled as nMPC-HY for this study) was undertaken by the prosthetist. During the tests, by changing the settings in the mobile software, the MPC-HY foot was separately adjusted in ascending and descending slope to achieve optimal walking comfort in each walking condition. In line with current practice, the FIX and nMPC-HY devices were set by the prosthetist for the most comfortable level ground walking experience and no change was made for slope ambulation. The ankle ROM setting in the MPC-HY foot is 5.2±0.8 degrees in plantar-flexion and 5.4±0.5 degrees in dorsi-flexion. In ascending slope, the ankle ROM setting in the nMPC-HY foot is 2.2±0.4 degrees in plantar-flexion and 8.8±0.4 degrees in dorsi-flexion, while in descending slope, the setting is 8.8±0.4 degrees in plantar-flexion and 2.2±0.4 degrees in dorsi-flexion respectively. The limb setup in the Linx system (subjects TF3 and TF5) was altered by the prosthetist to allow the knee and ankle to operate independently as conventional Orion knee and hydraulic ankle-foot devices respectively.

### Data collection protocol

Because of the similarity in nature of the study and the subjects, the protocol of this study was designed based on the method introduced by van der Linden et al. [16]. The subjects were asked to wear their common shoes and changed into shorts at the beginning of the tests. Since the FIX foot differed most from subjects’ normal prosthetic foot, it was used first by the amputees to maximise familiarisation time, as in addition to dedicated practice time with the new prosthetic components, subjects also stood/walked during the anthropometric measurement and marker placement. The alignment and adjustment of the prostheses was agreed by both the prosthetist and each subject to optimise the walking experience. Subjects were given time to practice walking in the laboratory until they felt safe and confident with the new prosthetic component. Anthropometric parameters were then measured with the FIX foot. The methods introduced by Goldberg were applied to measure the prosthetic segment parameters and the residual limb parameters for subsquent biomechanical modelling [17].

Fifteen markers (11 short base markers and 4 wand markers) were applied to the subject using a modified Helen Hayes marker placement. The short base markers were placed as follows: the sacrum (between the left and right posterior superior iliac spines), the ASIS (over the anterior superior iliac spines), the knee (laterally on the knee joint line on the intact side and laterally on the rotation centre on the prosthetic side), the ankle (on the lateral malleolus on the intact side; at the same horizontal level as the lateral malleolus on the shell and aligned with the centre line of the shank tube on the prosthetic side), and the toes (on the 1st metatarsal head and on the 5th metatarsal head on the intact side and the same nominal positions on the prosthetic side). The thigh wand marker was placed approximately in the middle between the greater trochanter and knee marker. The shank wand marker was placed approximately in the middle between the knee marker and lateral malleolus. The subjects were then asked to stand by a customised foot template, with the medial foot contacting two sides of the template, to align the thigh wand marker with the greater trochanter and knee marker and align the shank wand marker with the knee marker and lateral malleolus marker [18].

**Fig 1:**
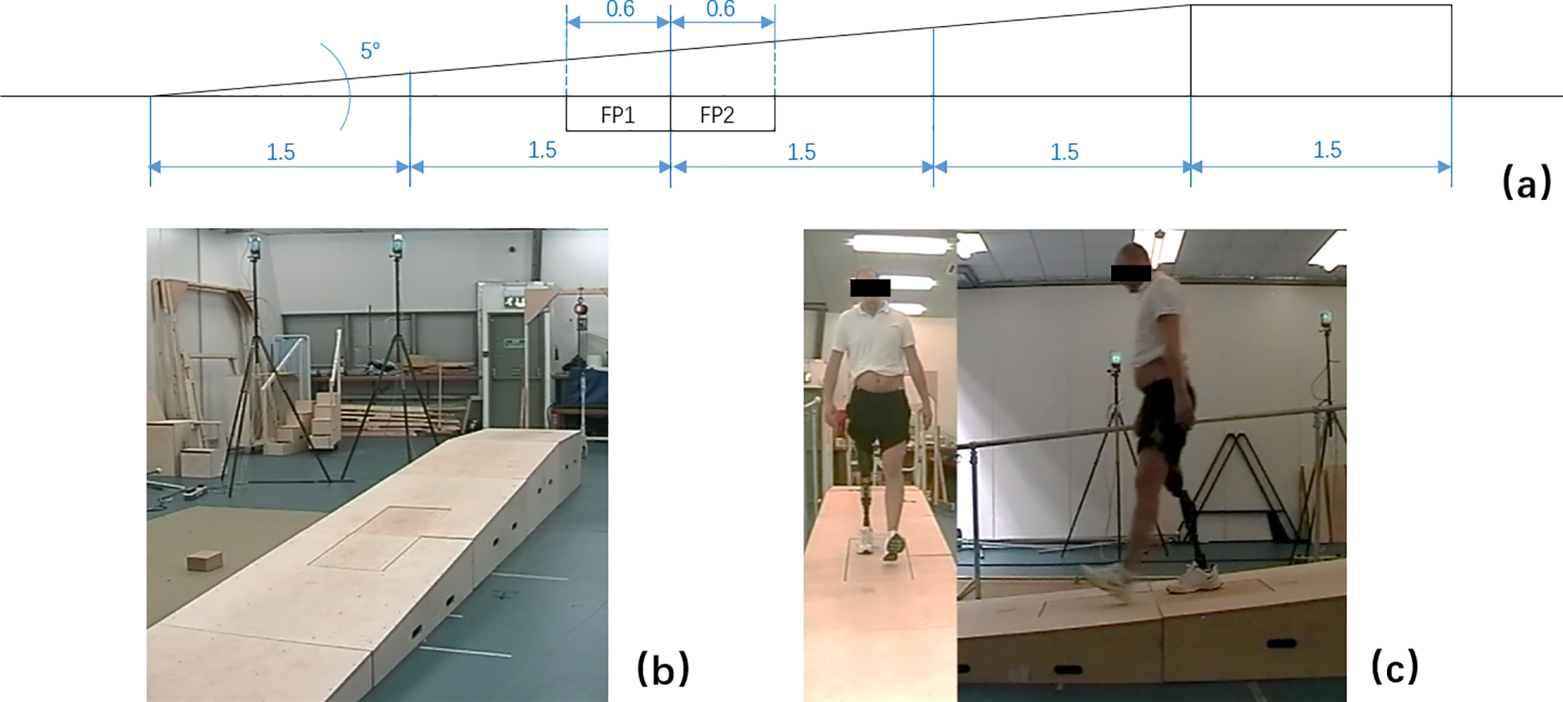
(a) Schematic of the slope design with major dimensions (unit: m). (b) Photograph of slope platforms and elements fitted with force plates. (c) One subject walking on the slope.

An 11-camera motion capture system (ProReflex, Qualisys AB, Sweden) with two force platforms (AMTI, USA, MODEL: BP400600HF-2000) were used to record the kinematic data (sampling at 120 Hz) and the ground reaction forces (GRFs, sampling at 240 Hz). The slope (5 degrees, 6 m in horizontal length of an inclined surface with 1.5 m level platform at the top end, 1 m in width) used in this study was designed based on Simon et al’s [19] concept to allow the floor level mounted force plates to record the GRFs on the slope surface. Fig 1 shows the schematic of the slope design showing the major dimensions together with photographs of the final setup of the walkway.

The subject was asked to ascend and descend the slope at a self-selected comfortable walking speed. Practise time was given and ground marks of appropriate start points were placed for each subject, so that the subject could make clean single foot contact with each force plate without posture adjustment. Five successful trials that recorded whole gait cycles (GCs) on both sides with complete kinematic data and ‘clean’ single foot contacts were collected for ascending and descending the slope respectively. The prosthetist then attached and adjusted the nMPC-HY and MPC-HY feet respectively for the subject and the test programme was repeated. Subjects were encouraged to have a rest between tests with the different prosthetic ankle-foot devices.

A questionnaire used in a previous study [20] to assess prosthetic ankles was answered by the subjects after the walking tests were completed with all prosthetic ankle-foot devices to provide a subjective assessment on the overall performance of the three ankle-foot device. The purposes of the questionnaire assessment were to check 1) if any differences between prosthetic ankle-foot devices were noted by subjects; 2) if there was any conflict between subject’s feedback and biomechanical assessment results; 3) if there was any issue with the prostheses that could not be indicated in the gait parameters measured in this research.

### Data processing

**Fig 2:**
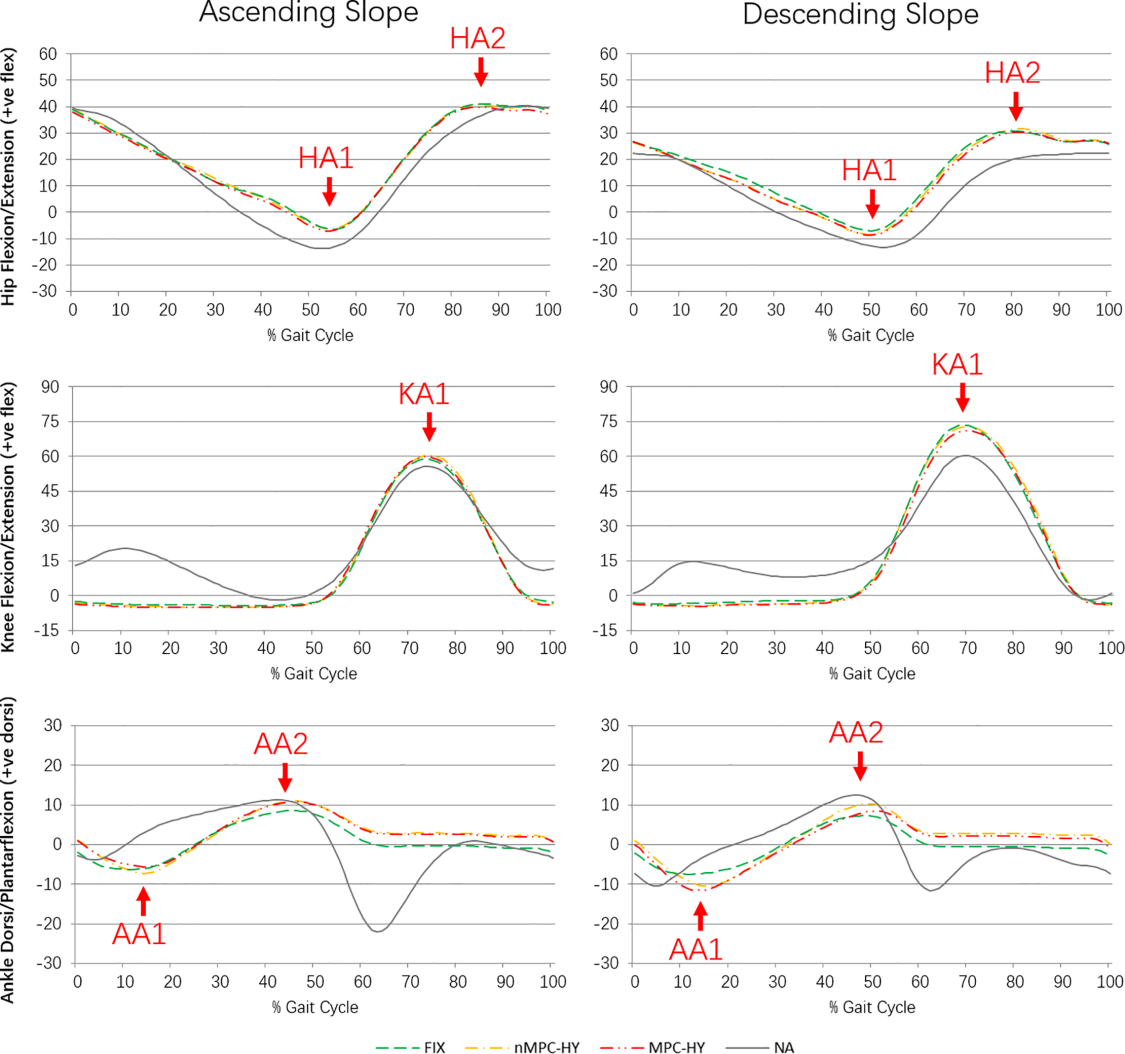
Mean curves of prosthetic side joint angles in the sagittal plane for ascending (left column) and descending (right column) a 5-degree slope. Unit: degrees.

**Fig 3:**
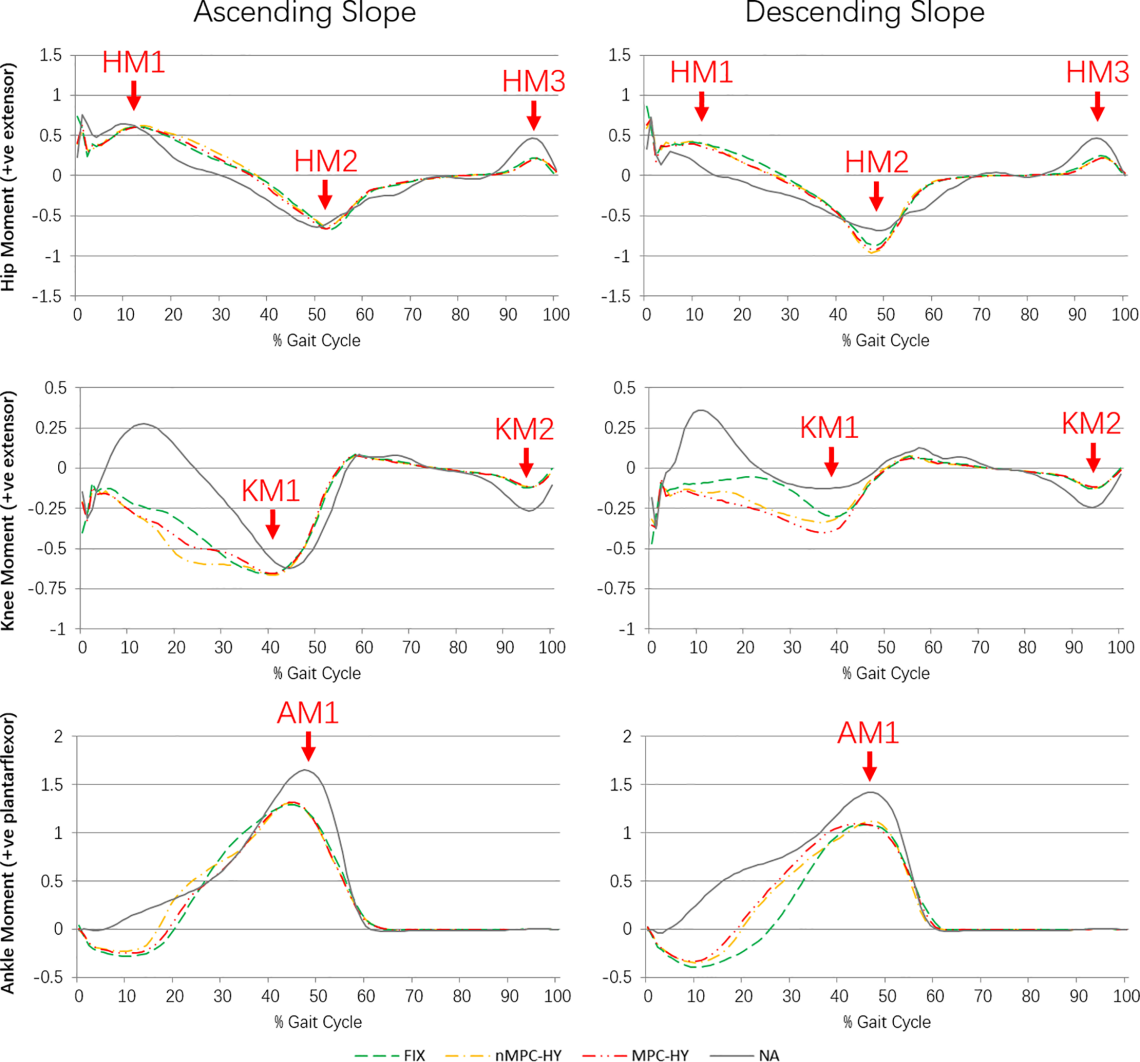
**Mean curves of prosthetic side lower joint moments in the sagittal plane in ascending (left column) and descending (right column) a 5-degree slope.** Unit: Nm/Kg.

The kinematic data was processed in Qualisys Track Manager (Qualisys, Sweden, version 2.6.682) to label the markers and then exported together with GRF data into Visual3D (C-Motion, USA, Student Edition, version 5.00.16) for further biomechanical modelling and relevant calculations. Gaps in the kinematic data that were no more than 10 frames were filled and the data were low pass filtered at 6 Hz (zero lag, 4th order, Butterworth filter). The GRF data was not filtered. Individually matched biomechanical models were generated for each prosthetic ankle-foot with the measured anthropometric data and prosthetic segment parameters [17]. The hip, knee and ankle joint angles in the sagittal plane were calculated and the ankle angles were normalised to the standing posture recorded with the foot template. This normalisation method helps to reduce the influence of different shoes used by subjects. The moments in the sagittal plane were computed using an inverse dynamics approach. The peak values (marked in Figs 2 and 3) during stance and swing phases were extracted from the GRFs, joint angles and moment waveforms for further analysis.

It has been reported that a more biomimicry prosthetic ankle moment pattern could introduce a substantial decrease in stresses on the residual limb [15]. The biomimicry of the ankle moment was generated by comparing the prosthetic ankle moment from each TFA subject with a mean ankle moment from the non-dominant side of NA group using a method known as normalcy TSI [21]. TSI compares two waveforms and returns a value between 0 and 1 to represent the level of similarity. A value of 1 represents perfect trend similarity between the two waveforms and a lower value indicates less similarity. To calculate normalcy TSI, ankle moments from each subject were firstly time-normalised to 101 points during the stance phase and all 101 points were used for comparison. This method has been introduced in the assessment of hydraulic ankle-foot devices and the calculation followed the steps described in a previous study [13].

Statistical analysis was applied to the extracted peak values and normalcy TSI values using IBM SPSS (IBM, USA, version 22.0.0.0). The Shapiro-Wilk test was applied to confirm the normalcy distribution of data. Significant differences between the different prosthetic ankle-foot devices (3 levels: FIX, nMPC-HY and MPC-HY) were determined by repeated measurements one-way analysis of variance (ANOVA) in ascending slope and descending slope respectively with post-hoc Tukey tests. The level of significance was set at p = 0.05.

## Results

### Walking speed and kinematics

The walking speed from each subject with each model of prosthetic ankle-foot device are provided in Table 2. There was no significant difference found in walking speed in either ascending slope (p = 0.993) or descending slope (p = 0.254) among the three models of prosthetic ankle-foot devices.

**Table 2:** Walking speed of each subject with each model of prosthetic ankle-foot device in ascending and descending slope. (unit: m/s).

ID	Ascending slope			Descending slope		
	FIX	nMPC-HY	MPC-HY	FIX	nMPC-HY	MPC-HY
TF1	1.37±0.03	1.28±0.05	1.25±0.05	1.11±0.06	1.14±0.02	1.10±0.05
TF2	1.05±0.06	1.12±0.02	1.19±0.03	1.28±0.05	1.30±0.07	1.23±0.07
TF3	0.85±0.05	0.84±0.03	0.85±0.05	1.12±0.07	1.11±0.10	1.05±0.02
TF4	1.04±0.04	1.00±0.05	1.02±0.05	1.06±0.02	1.00±0.05	1.07±0.02
TF5	1.07±0.02	1.11±0.03	1.13±0.06	1.05±0.05	1.07±0.05	1.06±0.06
Average	1.08±0.20	1.08±0.16	1.08±0.16	1.10±0.10	1.11±0.11	1.10±0.08
NA	1.18±0.14			1.14±0.16*		

*The subjects in the non-amputee group were requested to maintain a self-selected constant speed when descending slope.

The mean curves of the prosthetic side joint angles in the sagittal plane from the 5 TFA subjects are shown in Fig 2 (the data for the intact side is provided in the supporting information), together with the mean data from the NA group. Table 3 gives the details of the peak values (as marked in Fig 2) in the joint angles and p-values of one-way repeated measures ANOVA. The post-hoc results are described in the text when significant differences were found between the prosthetic ankle-foot devices.

**Table 3:** Peak values in the sagittal plane kinematic waveforms summarised from the 5 trans-femoral subjects and the one-way ANOVA results that compare the different prosthetic ankle-foot devices in ascending and descending the slope respectively. (unit: degrees; P: prosthetic side; I: intact side).

Peak point (point-side)	Ascending slope				Descending slope			
	FIX	nMPC-HY	MPC-HY	p value	FIX	nMPC-HY	MPC-HY	p value
HA1-P	-6.9±2.6	-6.8±3.1	-7.6±2.8	0.303	-7.7±2.4	-8.6±2.3	-9.2±3.3	0.341
HA1-I	-8.8±8.0	-10.1±7.7	-10.1±8.5	0.683	-9.6±4.0	-12.0±4.5	-11.7±4.4	0.050*
HA2-P	42.1±4.0	42.3±5.1	41.5±4.2	0.583	31.7±4.0	32.3±3.7	31.3±2.7	0.340
HA2-I	45.5±4.4	45.8±4.6	46.0±5.3	0.765	32.7±4.2	30.4±4.5	30.2±4.9	0.006*
KA1-P	60.0±8.7	61.4±8.3	61.2±7.3	0.409	74.3±5.9	73.2±5.8	71.8±6.5	0.218
KA1-I	52.3±3.7	51.0±4.2	52.4±3.8	0.236	61.2±3.5	61.9±3.1	61.8±2.3	0.586
AA1-P	-6.4±2.9	-7.5±1.8	-6.0±2.7	0.307	-7.5±2.9	-10.7±1.8	-12.0±2.4	0.001*
AA1-I	-2.0±6.0	-0.7±6.2	-0.8±6.4	0.624	-12.3±4.8	-10.6±5.0	-10.6±5.5	0.330
AA2-P	8.6±2.1	11.1±2.9	10.9±2.6	0.005*	7.6±2.4	10.4±3.1	8.8±2.9	0.011*
AA2-I	6.3±2.5	8.0±3.3	8.2±4.5	0.581	9.5±3.7	9.1±3.3	10.0±4.7	0.882

* p value ≦ 0.05

In the ascending slope, significant differences were observed in the prosthetic side maximum dorsiflexion (AA2-P), where the two hydraulic ankles showed significantly greater dorsiflexion angle than the fixed ankle (nMPC-HY vs FIX: p = 0.003; MPC-HY vs FIX: p = 0.005; nMPC-HY vs MPC-HY: p = 0.738).

In the descending slope, significant differences were found in the intact side hip angles (maximum extension and flexion) and prosthetic side ankle angles (maximum dorsiflexion and plantarflexion). In detail, on the intact side, when the hydraulic ankles were used, there was a greater maximum hip extension (HA1-I; nMPC-HY vs FIX: p = 0.027; MPC-HY vs FIX: p = 0.040; nMPC-HY vs MPC-HY: p = 0.797) and a reduced maximum hip swing flexion (HA2-I; nMPC-HY vs FIX: p = 0.006; MPC-HY vs FIX: p = 0.003; nMPC-HY vs MPC-HY: p = 0.665). On the prosthetic side, there was significantly increased maximum plantarflexion in the hydraulic ankles (AA1-P; nMPC-HY vs FIX: p = 0.003; MPC-HY vs FIX: p<0.001; nMPC-HY vs MPC-HY: p = 0.124). The nMPC-HY showed a significantly higher dorsiflexion angle (AA2-P) than the other two prosthetic ankle-foot devices (nMPC-HY vs FIX: p = 0.003; nMPC-HY vs MPC-HY: p = 0.047), while the difference between the FIX and MPC-HY (MPC-HY vs FIX: p = 0.119) was not statistically significant.

### Kinetics

The mean curves of the prosthetic side sagittal plane joint moments from the 5 TFA subjects are shown in Fig 3 (the data for the intact side is provided in the supporting information), together with the mean NA data for reference. Table 4 gives the details of peak values (as marked in Fig 3) in the joint moment patterns and p-values of one-way repeated measures ANOVA.

**Table 4:** Peak values in the sagittal plane kinetic waveforms summarised from the 5 trans-femoral subjects and the one-way ANOVA results that compare different prosthetic ankle-foot devices in ascending and descending the slope respectively. (unit: Nm/Kg; P: prosthetic side; I: intact side).

Peak point (point-side)	Ascending slope				Descending slope			
	FIX	NMPC-HY	MPC-HY	p value	FIX	NMPC-HY	MPC-HY	p value
HM1-P	0.62±0.20	0.64±0.19	0.64±0.18	0.662	0.44±0.14	0.46±0.16	0.42±0.14	0.519
HM1-I	1.14±0.12	1.15±0.12	1.13±0.05	0.947	0.52±0.16	0.64±0.25	0.49±0.25	0.608
HM2-P	-0.71±0.28	-0.69±0.25	-0.71±0.26	0.378	-0.95±0.33	-0.99±0.35	-0.97±0.34	0.108
HM2-I	-0.33±0.17	-0.24±0.23	-0.23±0.17	0.177	-0.55±0.17	-0.57±0.17	-0.58±0.13	0.271
HM3-P	0.22±0.14	0.22±0.10	0.22±0.08	0.964	0.25±0.05	0.32±0.07	0.23±0.06	0.130
HM3-I	0.83±0.20	0.74±0.17	0.82±0.16	0.343	0.94±0.19	0.90±0.20	0.94±0.21	0.484
KM1-P	-0.67±0.12	-0.69±0.07	-0.68±0.11	0.782	-0.33±0.10	-0.34±0.09	-0.41±0.09	0.032*
KM1-I	-0.85±0.17	-0.87±0.19	-0.86±0.11	0.839	-0.43±0.22	-0.46±0.22	-0.43±0.23	0.462
KM2-P	-0.19±0.22	-0.12±0.04	-0.12±0.03	0.479	-0.19±0.13	-0.13±0.02	-0.12±0.02	0.299
KM2-I	-0.35±0.11	-0.37±0.09	-0.41±0.09	0.274	-0.39±0.12	-0.40±0.08	-0.42±0.08	0.519
AM1-P	1.30±0.06	1.33±0.08	1.34±0.10	0.185	1.10±0.12	1.13±0.15	1.10±0.11	0.433
AM1-I	1.61±0.10	1.65±0.08	1.64±0.10	0.055	1.38±0.12	1.42±0.10	1.40±0.08	0.367

* p value ≦ 0.05

For the ascending slope data, there was no significant difference in the kinetic peak values. In the descending slope, a significant difference was noted in the prosthetic side maximum external knee extensor moment (KM1-P), where a significantly higher flexor moment was found at the prosthetic knee joint when the MPC-HY was used (nMPC-HY vs FIX: p = 0.764; MPC-HY vs FIX: p = 0.017; nMPC-HY vs MPC-HY: p = 0.027).

### Normalcy TSI of the ankle moment

For ascending slope, a significant difference was found in the normalcy TSI of the prosthetic ankle moment (FIX: 95.0±1.4%; nMPC-HY: 95.9±0.6%; MPC-HY: 96.8±0.7%; p = 0.008), where the MPC-HY showed a significantly higher TSI value than the FIX (nMPC-HY vs FIX: p = 0.062; MPC-HY vs FIX: p = 0.002; nMPC-HY vs MPC-HY: p = 0.059).

For descending slope, a significant difference was also found in the normalcy TSI of the prosthetic ankle moment (FIX: 92.1±2.0%; nMPC-HY: 94.7±0.7%; MPC-HY: 95.3±0.9%; p = 0.002) and both of the hydraulic devices showed a significantly higher normalcy TSI than the FIX (nMPC-HY vs FIX: p = 0.003; MPC-HY vs FIX: p = 0.001; nMPC-HY vs MPC-HY: p = 0.353).

### Questionnaire

Table 5 shows the questions used in the questionnaire and summarises the results of the rating by the 5 TFA subjects. In general, subjects considered that the hydraulic foot offered improvements over the non-hydraulic foot. However, the differences between the nMPC-HY and MPC-HY was not notable. Two subjects highlighted improved safety with the hydraulic ankles especially in descending slope in the additional comment area at the end of the questionnaire.

**Table 5:** Results from the questionnaire that assessed the overall performance of the prosthetic ankle-foot devices by the 5 TFA subjects. (1: strongly disagree; 2: disagree; 3: neutral; 4: agree; 5: strongly agree).

Questions	FIX	nMPC-HY	MPC-HY
1. The current ankle adds noticeable weight to my prosthesis.	2.2±1.1	2.8±1.5	2.8±1.5
2. If I have pain in my residual limb, this ankle reduces it.	1.4±0.5	4.0±0.8*	4.3±1.2**
3. This ankle increases comfort during walking.	1.2±0.4	4.4±0.5	4.6±0.5
4. This ankle makes my prosthesis harder to swing as I walk.	2.6±1.1	1.8±0.4	2.0±1.2
5. This ankle enables me to walk longer distances.	1.2±0.4	4.4±0.5	4.2±0.8
6. This ankle increases the effort to walk.	4.8±0.4	1.4±0.5	1.6±0.5
7. I am able to walk faster with this ankle.	2.0±1.0	4.4±0.5	4.2±0.8
8. Walking feels smoother with this ankle.	1.2±0.4	4.6±0.5	4.6±0.5
9. This ankle makes me feel like I am stepping into a hole.	1.8±1.0*	2.0±1.2*	1.5±1.0*
10. This ankle reduces twisting between my socket and residual limb.	1.6±0.9	3.8±0.8	3.6±0.9
11. This ankle increases my comfort during standing.	1.2±0.4	4.0±1.2	3.6±1.7
12. This ankle decreases stability during standing.	4.2±0.8	2.6±1.8	2.0±1.7
13. This ankle makes me feel unstable during walking.	4.0±0.7	1.4±0.5	1.2±0.4
14. This ankle allows me to be more active.	1.4±0.5	4.2±0.8	3.6±1.5
15. This ankle enables me to turn easier.	1.6±0.9	4.2±0.8	4.0±1.0
16. It is easier for me to walk up an incline with this ankle.	2.0±1.2	4.4±0.5	4.8±0.4
17. It is easier for me to walk down an incline with this ankle.	1.0±0.0	4.4±0.5	4.6±0.5
18. This ankle makes it easier for me to walk on uneven ground.	1.6±0.9	4.4±0.5	4.6±0.5
19. This ankle provides too much motion.	1.4±0.9	1.4±0.5	1.4±0.9
20. This ankle doesn’t provide enough motion.	4.6±0.9	1.2±0.4	2.4±1.3
21. This ankle makes my prostheses feel less rigid.	1.2±0.4	3.2±1.5	3.2±1.6
22. This ankle makes me feel like I’m walking up hill.	2.5±1.9*	1.8±1.0*	2.0±1.2*
23. This ankle makes me feel like I’m walking down hill.	3.5±2.0*	1.8±1.0*	2.0±1.2*
24. This ankle makes me stub my toe more during swing.	2.8±0.5	2.0±1.0	1.6±0.9
25. Overall, this ankle provides me with greater comfort.	1.4±0.5	4.4±0.5	4.4±0.5
26. I like having this ankle in my prosthesis.	1.0±0.0	4.8±0.4	4.8±0.4

* One subject rated NA (not applicable) in this question.

** Two subjects rated NA (not applicable) in this question.

## Discussion

The first aim of this study was to assess the hydraulic ankle-foot devices in ascending and descending slope with TFAs. The second aim of this study was to investigate the effects of MPC-HY foot in adaption to a slope ambulation. In general, the major benefits of the hydraulic devices, compared with the FIX, are considered to be the improved biomimicry in prosthetic ankle moment patterns and the greater ROM for slope adaption as hypothesised. In addition, the hydraulic devices were found to be able to enhance the walking safety in descending slope by helping maintain prosthetic knee stability, which will be discussed later. The use of the micro-processor allowed the subject to achieve optimised setting of prosthetic ankle resistance and ROM to adapt to inclined surfaces. The optimisation resulted in the differences in prosthetic ankle angle and moment patterns that can be observed from Fig 2 and Fig 3. However, in most of the assessed parameters, the differences between MPC-HY and nMPC-HY do not reach a statistically significant level.

A higher normalcy TSI value of the prosthetic ankle moment indicates a better similarity between prosthetic and biological patterns. In both ascending and descending slope, the MPC-HY showed a significantly higher prosthetic ankle moment TSI than the FIX. The nMPC-HY also provided significantly higher normalcy TSI than the FIX in descending slope (p = 0.003), and the p value in ascending slope is very close to the statistically determinant significance level (p = 0.062). This result generally agrees with a previous study that investigated the performance of a hydraulic ankle-foot device, in which the nMPC-HY was found to be able to create a significantly higher normalcy TSI in the prosthetic ankle moment than the FIX across a range of level and camber ground conditions [13]. It can be noticed from the ankle moment in ascending slope (see Fig 3 left column third row) that during the dorsiflexion period (from about 25% to 45% of GC, the ankle moves from a neutral position to near maximum dorsiflexion), the hydraulic ankles had concave resistance patterns that are similar to the biological curve exhibited by the NA group, while the FIX showed a convex pattern as in a conventional rigid ankle [15, 22, 23]. A similar difference in the prosthetic ankle moment patterns between nMPC-HY and FIX was also observed from the figures reported in a previous study with the TTAs [9]. Pitkin presented a study with a mathematical model that linked the prosthetic ankle resistance moment with the stresses on the residual limb in the TTAs, which reported a substantial decrease in residuum stresses when a bio-mimicing ankle moment pattern was generated [15]. TFAs were hypothesised to be able to receive similar benefits from biologically compliant prosthetic ankles and knees [15]. Therefore, with a more bio-mimicing ankle moment pattern, the hydraulic ankle theoretically improved the socket comfort for prostheses users. However, there was not a consistent conclusion from the two previous studies that analysed the internal stresses applied to the residual limb in the TTAs when comparing nMPC-HY with other rigid ankle-foot devices, as one study reported significantly reduced internal stresses at residual limb [7] while the other study reported no significant difference in the torque at the distal end of the prosthetic socket [14]. In this study, the questionnaire included a question relating to the internal stresses (Table 5 question 10) and the subjects considered that the hydraulic ankles helped to reduce the socket-stump twisting, which could support Pitkin’s hypothesis with TFAs. A future study that directly monitors the internal stresses in the socket is suggested to confirm this benefit from the hydraulic ankle-foot devices.

The ankle joint ROM was determined from the maximum ankle angles during stance in this study. Except for the maximum plantarflexion (AA1-P) in ascending slope (probably due to the uphill ground condition that reduced the requirement for the maximum plantarflexion), the hydraulic devices reached significantly greater peak ankle angles compared with the FIX. Therefore, it was generally considered that the hydraulic ankle devices could adapt to a slope surface better by providing a larger ROM as required. This finding was also supported by the subjects’ feedback in the questionnaire (Table 5 question 16 and 17). In the previous study on level and camber walking, nMPC-HY had also been found to be able to produce a significantly higher maximum dorsiflexion and plantarflexion angle compared to the FIX [13].

Another improvement of the hydraulic ankles was found in the prosthetic knee moment during downhill walking. As shown in Fig 3 (second row right column), a very low internal knee flexor moment (equals to the external knee extensor moment) close to zero on the prosthetic side occurred at about 24% of GC when the FIX was used, while the hydraulic ankles facilitated a greater knee flexor moment during the whole mid-stance phase. This could be an explanation for the comments from the subjects regarding the strongly improved feeling of safety with the hydraulic ankles in downhill walking in this study. The passive prosthetic knee remains in maximum extension during mid-stance (Fig 2 middle row), therefore the internal knee flexor moment is a reaction moment to neutralise the extension moment created by the external forces. As the passive knee could not actively provide an extensor moment, if the external loads cause a flexion moment to the knee joint during mid-stance, there is a risk of knee collapse, which is more likely to happen in descending slope compared to level walking. So it is believed that subject’s experience on downhill walking safety is affected by the sensor feedback on knee moment and the hydraulic ankles therefore help to enhance the safety feeling. However, with limited experimental and theoretical study relevant to this finding, considering there might be other characteristics of the prosthetic ankle-foot device that caused the change in prosthetic knee moment, such as the location difference in simulated ankle rotation axis, future work is suggested to confirm the specific feature in the prosthetic ankle-foot device that is linked to the prosthetic knee moment.

The difference between the two hydraulic ankle-foot devices was the application of a micro-processor, which allowed the MPC-HY to realise customised setting for slope adaption. The timing (as a percentage of GC) when the prosthetic ankle reached maximum plantarflexion, neutral position, and maximum dorsiflexion was generally the same between the two hydraulic devices (Fig 2 third row), while the difference in the prosthetic ankle moments during the dorsiflexion progress can be observed from the corresponding curves. When walking up a slope, as illustrated in Fig 3 (third row left column), from about 15% to 32% of GC, the MPC-HY provided less plantarflexor moment compared with nMPC-HY to make rolling at the ankle joint easier. The plantarflexor moment then increased to be the same as nMPC-HY to support lifting the body up and through push-off. When walking down the slope, as expected, the MPC-HY foot showed higher plantarflexor moments during the entire roll-over process. MPC-HY reached the maximum plantarflexor moment earlier than nMPC-HY and maintained the maximum plantarflexor moment for a longer period. This corresponds to the reduced maximum dorsiflexion angle (AA2-P) found in the MPC-HY compared to the nMPC-HY (p = 0.047). The MPC-HY showed higher normalcy TSI in the prosthetic ankle moment of the three ankle-foot devices in both ascending and descending slope, which potentially indicated a better mimicry of a biological ankle function. MPC-HY also enabled a significantly greater maximum external knee extensor moment (KM1-P) than the nMPC-HY in descending slope (p = 0.027). Therefore, it is considered that the customisation of settings helped to enhance the benefits of the hydraulic ankle. However, despite the improvements observed in the kinetic patterns and some peak values, the difference between the MPC-HY and nMPC-HY was not very notable by subjects according to the questionnaire results. This was probably because the conservative activities tested in this project were readily accomplishable by the nMPC-HY foot without changing the plantarflexor moment. A steeper slope might have better demonstrated the performance of MPC-HY foot. One advantage of the real time control function is that the TFAs can adjust the prostheses to adapt to different shoes and ground conditions (e.g. increase the stiffness of ankle for a wet and slippery surface) without assistance from prosthetist, which was considered to improve the convenience of using prostheses and life quality. Therefore, for the prosthesis user, the choice between MPC-HY and nMPC-HY should be based on individual demands such as outdoor activities and the required frequency of adjustment in prostheses.

The statistical tests used in this research were not based on a prior assessment of a clinically significant change. This was because a definition of a clinically significant difference for the primary outcome measure (ankle moment TSI) was not available in the literature. Besides, the gait of amputees is formed during their rehabilitation programme and often will not be changed to reach a clinically significant difference in a short period. In this research all subjects finished their tests in a single experiment day whereas a number of the studies on prosthetic component evaluation allow only up to 4 weeks fitting and acclimatisation. Furthermore, most subjects that have been recruited to the studies that test different prosthetic components are good walkers which may limit room for a clinically significant improvement. However it is anticipated that the results from this work can be used to help inform (power) calculations to determine the number of volunteers required for future studies.

The main limitation of this study is that the research approach is more commonly used in NA, while the differences between NA and TFA is not negligible. The chosen marker set in this research is normally applied to the NA. Unlike specific running prostheses, where the markers can be directly placed on the mechanical structure, most of the mechanical structure in conventional prosthetic ankle-foot devices is covered by the foot shell and shoes during the walking. The detailed motion and deformation of the underlying mechanical structure therefore is not recorded or observed. In most of the kinematic studies in prosthetics, the markers on the prosthetic side are placed in the same positions as the intact side, accepting, for example, that the actual rotation centre of the prosthetic ankle joint is not exactly the same as that of the biological ankle. Another limitation of this research is the modelling method used in the inverse dynamics calculation. Some assumptions made in the conventional inverse dynamics approach, such as the rigid body assumption, were not entirely applicable to the prostheses. In addition, although the segment parameters on the prosthetic side were measured separately, some compromises had to be made during modelling. For example, the hydraulic body above the ankle rotation axis cannot be separated from the “foot” part, therefore the centre of mass and moments of inertia of the prosthetic shank segment cannot be directly measured. Therefore the prosthetic foot segment parameters in the hydraulic devices was considered to be the same as the FIX and the hydraulic body was modelled as a rigid cylinder with uniform density when calculating the prosthetic shank segment parameters. Some prostheses-specific models have been developed to calculate prosthetic ankle power in energy storing foot devices [24], however, to the authors’ knowledge, so far there is no published work that reports an improved calculation of prosthetic ankle moments.

There are some other potential issues in the experiment design.The TFA subjects who participated in this study are experienced and able walkers who were considered able to adapt to new prosthetic ankle-foot devices in a relatively short period. So the tests with different prosthetic ankle-foot devices were finished in one day for each subject and this helped to reduce the potential errors caused by marker displacement and environment change. However, long term use of a different prosthetic component may further change the gait of patients and there is little research on the accommodation time of prostheses [25]. Additionally, the test sequence of the three prosthetic ankle-foot devices was designed to maximise the practise time for the foot that differed most from the subject’s common prosthesis. A random test sequence may better reflect the result. Fatigue is normally considered as a problem for a unified test sequence. However considering sufficient rest time was given to the subjects in this research, the test sequence should not be a serious issue The GRF data was not filtered therefore the noise may influence the result. In the calculation of normalcy TSI, a reference group with gender and age matching subjects and controlled speeds is suggested. Although subjects were given more time to practice with the FIX device and did not use their usual prosthetic ankle-foot devices during the test, 3 of the 5 subjects’ usual prostheses contain hydraulic ankle-foot devices, which may influence the results of this study. Besides, in order to reduce the fitting time, subjects used their usual prosthetic knee during the test, which may also affect the results. Considering the non-blind experimental design and the use of an invalidated questionnaire, the results from the questionnaire were only used to provide additional interpretation of comparative biomechanical performance for the three prosthetic ankle-foot devices.

A relatively small number of subjects participated in this study, therefore the capability of the statistical method (repeated measures ANOVA) is insufficient to reach a conclusive determination and the statistical results in this study are intended to provide indicative analysis only. Further study with an increased number of TFA subjects is required to test the findings. Involvement of other brands and models of hydraulic ankle-foot device is needed to confirm the usual features of hydraulic ankles.

## Conclusion

In conclusion, according to the statistical results, the hydraulic ankle-foot devices (with or without micro-processor control) showed a relatively better bio-mimicking of the ankle moment, a slightly increased ROM, and enhanced walking safety (preventing knee collapse) compared to the FIX in 5 degrees slope ambulation with TFAs. The MPC-HY permits customised changes in the hydraulics and ROM settings for ascending and descending slope respectively, however, the quantified differences found in the data between the MPC-HY and nMPC-HY were not generally perceived by the subjects. Overall, it is suggested that the hydraulic ankle-foot devices show a potential to provide better and safer adaption to slope surfaces, which may improve the walking experience of active TFAs with outdoor walking requirements. The micro-processor controlled device is perhaps more compatible with users that have demands for frequent adjustment of their prosthetic ankle resistance and ROM, e.g. in more demanding conditions (e.g. slope inclination/declination) than used in this study.

## Supporting information

S1 Fig **Mean curves of intact side joint angles in the sagittal plane for ascending (left column) and descending (right column) a 5-degree slope**. Unit: degrees.
(TIF)

S2 Fig **Mean curves of intact side lower joint moments in the sagittal plane in ascending (left column) and descending (right column) a 5-degree slope.** Unit: Nm/Kg.
(TIF)

S1 Dataset Time-normalised sagittal plane lower joint (hip/knee/ankle) angles and moments from five trans-femoral amputee subjects. A set of five trails from each subject in two walking conditions (ascending and descending a 5-degree slope) with three types of prosthetic ankle/foot (Echelon/Esprit/Elan). All data have been time-normalised to 101 points over stance phase and exported to excel files.
(ZIP)
