# Supplementary figures and images for: A biomechanical assessment of hydraulic ankle-foot devices with and without micro-processor control during slope ambulation in trans-femoral amputees
Source: PLoS One. 2018 Oct 5;13(10):e0205093. doi: 10.1371/journal.pone.0205093 (PMC6173401; doi:10.1371/journal.pone.0205093)

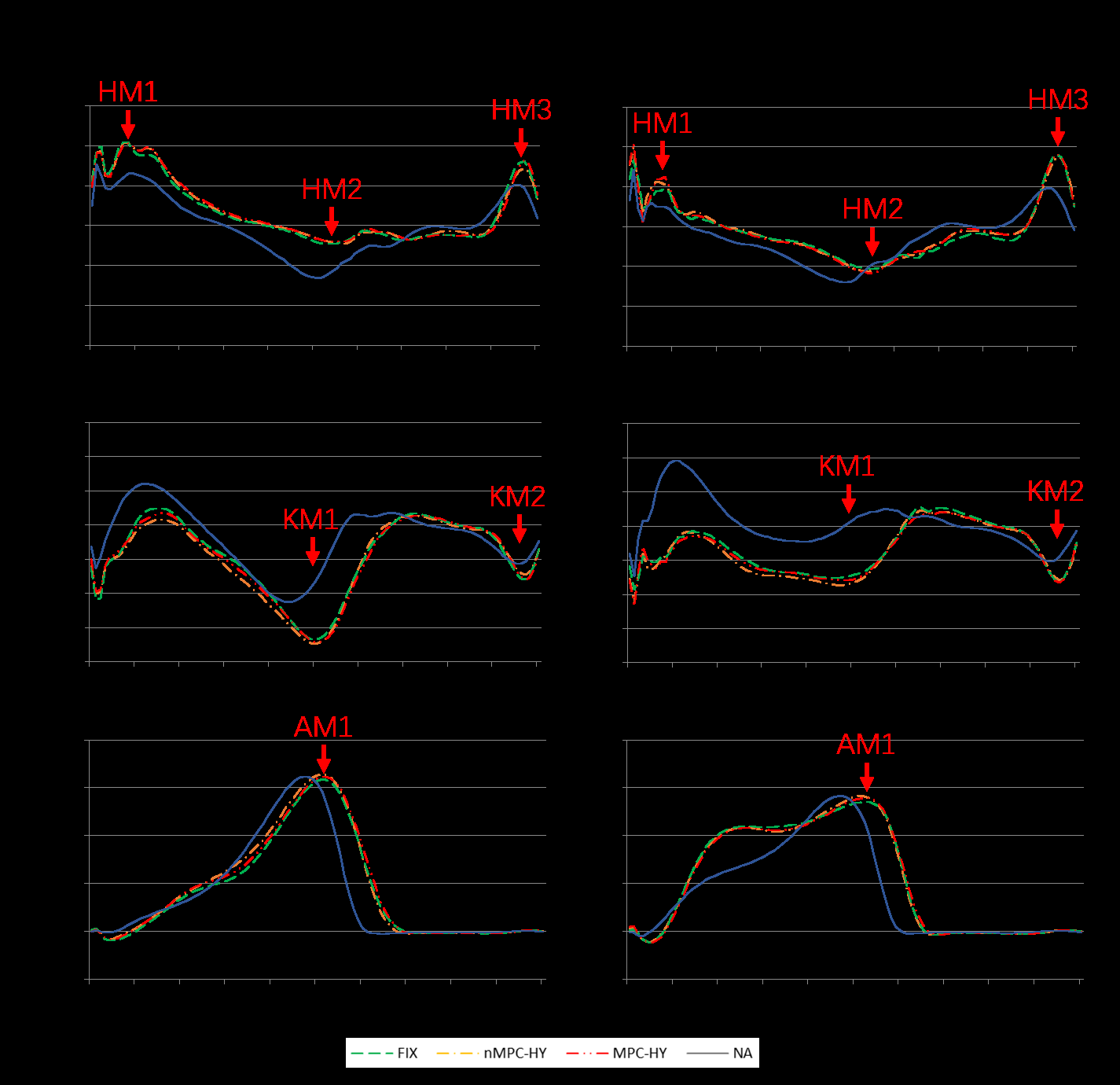

Supplement: S1 Fig — Mean curves of intact side joint angles in the sagittal plane for ascending (left column) and descending (right column) a 5-degree slope. Unit: degrees. (TIF) [file pone.0205093.s001.tif]
